# Chronic Pain Treatment Strategies in Parkinson’s Disease

**DOI:** 10.3390/neurolint12030014

**Published:** 2020-11-18

**Authors:** Amber Edinoff, Niro Sathivadivel, Timothy McBride, Allyson Parker, Chikezie Okeagu, Alan D. Kaye, Adam M. Kaye, Jessica S. Kaye, Rachel J. Kaye, Meeta M. Sheth, Omar Viswanath, Ivan Urits

**Affiliations:** 1 Department of Psychiatry and Behavioral Medicine, Health Science Center, Louisiana State University Shreveport, Shreveport, LA 71103, USA; nsath1@lsuhsc.edu; 2 School of Medicine, Louisiana State University Shreveport, Shreveport, LA 71103, USA; tmcbri@lsuhsc.edu (T.M.); apar13@lsuhsc.edu (A.P.); 3 Department of Anesthesiology, Louisiana State University New Orleans, New Orleans, LA 70112, USA; cokeag@lsuhsc.edu (C.O.); akaye@lsuhsc.edu (A.D.K.); 4 Department of Anesthesiology, Louisiana State University Shreveport, Shreveport, LA 71103, USA; mshet3@lsuhsc.edu (M.M.S.); viswanoy@gmail.com (O.V.); ivanurits@gmail.com (I.U.); 5 Department of Pharmacy Practice, Thomas J. Long School of Pharmacy and Health Sciences, University of the Pacific, Stockton, CA 95211, USA; akaye@pacific.edu (A.M.K.); j_kaye1@u.pacific.edu (J.S.K.); 6 School of Medicine, Medical University of South Carolina, Charleston, SC 29425, USA; rachelkaye17@hotmail.com; 7 College of Medicine-Phoenix, University of Arizona, Phoenix, AZ 85004, USA; 8 Department of Anesthesiology, School of Medicine, Creighton University, Omaha, NE 68124, USA; 9 Valley Anesthesiology and Pain Consultants–Envision Physician Services, Phoenix, AZ 85004, USA; 10 Southcoast Health, Southcoast Physicians Group Pain Medicine, Wareham, MA 02571, USA

**Keywords:** chronic pain, Parkinson’s disease, treatment strategies, neuropathic pain

## Abstract

Neurological disorders, including Parkinson’s disease (PD), have increased in prevalence and are expected to further increase in the coming decades. In this regard, PD affects around 3% of the population by age 65 and up to 5% of people over the age of 85. PD is a widely described, physically and mentally disabling neurodegenerative disorder. One symptom often poorly recognized and under-treated by health care providers despite being reported as the most common non-motor symptom is the finding of chronic pain. Compared to the general population of similar age, PD patients suffer from a significantly higher level and prevalence of pain. The most common form of pain reported by Parkinson’s patients is of musculoskeletal origin. One of the most used combination drugs for PD is Levodopa-Carbidopa, a dopamine precursor that is converted to dopamine by the action of a naturally occurring enzyme called DOPA decarboxylase. Pramipexole, a D2 dopamine agonist, and apomorphine, a dopamine agonist, and Rotigotine, a dopamine receptor agonist, have showed efficacy on PD-associated pain. Other treatments that have shown efficacy in treating pain of diverse etiologies are acetaminophen, Nonsteroidal anti-inflammatory drugs (NSAIDs), and cyclooxygenase-2 (COX-2) inhibitors. Opioids and opioid-like medications such as oxycodone, morphine, tramadol, and codeine are also commonly employed in treatment of chronic pain in PD. Other opioid related medications such as Tapentadol, a central-acting oral analgesic with combined opioid and noradrenergic properties, and Targinact, a combination of the opioid agonist oxycodone and the opioid antagonist naloxone have shown improvement in pain. Anticonvulsants such as gabapentin, pregabalin, lamotrigine, carbamazepine and tricyclic antidepressants (TCAs) can be trialed when attempting to manage chronic pain in PD. The selective serotonin and noradrenaline reuptake inhibitors (SNRIs) also possess pain relieving and antidepressant properties, but carry less of the risk of anticholinergic side effects seen in TCAs. Deep brain stimulation (DBS) of the subthalamic nucleus (STN) has been shown in multiple studies to be effective against various types of PD associated pain symptoms. Massage therapy (MT) is one of the most common forms of complementary and alternative medicine. Studies have shown that pressure applied during MT may stimulate vagal activity, promoting reduced anxiety and pain, as well as increasing levels of serotonin. In a survey study of PD patients, rehabilitative therapy and physical therapy were rated as the most effective for pain reduction, though with only temporary relief but these studies were uncontrolled. Yoga has been studied for patients with a wide array of neurological disorders. In summary, PD pathology is thought to have a modulating effect on pain sensation, which could amplify pain. This could help explain a portion of the higher incidence of chronic pain felt by PD patients. A treatment plan can be devised that may include dopaminergic agents, acetaminophen, NSAIDs, opioids, antidepressants, physical therapies, DBS and other options discussed in this review. A thorough assessment of patient history and physical examination should be made in patients with PD so chronic pain may be managed effectively.

## 1. Introduction

The continually industrializing world has introduced major medical advancements that have significantly improved the quality of health care we now possess. As a result, there are more people alive today and living longer than ever before. However, such progress does not develop without further challenge, as we now see neurological disorders becoming the primary cause of disability, of which Parkinson’s disease (PD) is the fastest growing with a doubling in cases between 1995 and 2015 (to 6 million) and another doubling projected by 2040 [1]. As age-related illnesses such as PD increase in prevalence, it should be of primary concern to a new generation of health care providers to equip themselves with the necessary knowledge and tools to diagnose and treat them.

Parkinson’s disease is a widely described, physically and mentally disabling neurodegenerative disorder that is most often recognizable in patients by the presence of three cardinal motor signs: resting tremor, bradykinesia, and muscular rigidity. Any one of these symptoms are present in 70–90% of Parkinson’s patients and allow for a good diagnostic potential [2]. Accompanying the motor symptoms are the non-motor presentations of PD, such as depression, insomnia, and cognitive decline, all of which are well-known to negatively affect the quality of life in the Parkinson’s patient [3,4]. One symptom often poorly recognized and under-treated by health care providers despite being reported as the most common non-motor symptom is the finding of chronic pain [5,6,7,8]. Chronic pain was noted in PD since its original description in 1817 by James Parkinson in such ways as, “[…] great pain in both arms, extending from the shoulder to the finger ends. […] leaving both the arms and hands in a very weakened and trembling state.” Further, it continues to be a lesser-known characteristic of the disease in the greater number of Parkinson’s patients seen today [7,9]. While it is understood that some level of pain is caused and/or made worse by PD, the presentation of pain in patients is greatly varied in type and severity, with some patients reporting no pain and others experiencing several modalities of pain ranging from minor to moderate and severe [6,10]. If the health care provider is made better aware of the presentations of chronic pain in PD, then a diagnosis and effective treatment plan is more likely.

To relay our current understanding of chronic pain in Parkinson’s disease, this review will describe Parkinson’s epidemiology, pathophysiology, risk factors and presentation. It will define chronic pain and investigate the various modalities of chronic pain and some possible explanations for relation to PD pathology. Lastly, the management and current recommended therapies of chronic pain in PD will be discussed.

## 2. Parkinson’s Disease

### 2.1. Epidemiology

PD is the most common movement disorder and the second most common neurodegenerative disorder of the central nervous system. The disease affects around 3% of the population by age 65 and up to 5% of people over the age of 85 [11]. Estimates on the incidence and prevalence of PD vary across studies, which can likely be attributed to differences in methodology, means of data collection, patient survival, case ascertainment, and diagnostic criteria [12]. Most studies yield a prevalence of 100 to 200 per 100,000 people, with annual incidence estimates ranging from less than 10 to more than 20 per 100,000 in the total population [13]. However, these numbers increase precipitously with age, with a median incidence rate of 160 per 100,000 in the population aged 65 years or older and peaking in most studies between the ages of 70 and 79 years [14,15]. PD is rare prior to the age of 50 with onset before age 40 seen in less than 5% of cases in population-based cohorts [13]. Risk of PD is about 1.5 times higher in men than in women. [14]. A meta-analysis of international studies demonstrated that the incidence of PD is increasing in both men and women [15]. 

### 2.2. Pathophysiology and Genetics

PD is defined by loss of dopaminergic neurons in the substantia nigra with deposition of intraneuronal aggregates composed of α-synuclein, called Lewy bodies, in affected areas. Lewy body pathology in PD not only involves cells that produce dopamine, but also those responsible for producing acetylcholine, norepinephrine, serotonin, histamine, and glutamate, accounting for its wide spectrum of clinical symptoms [16]. The primary cause and the mechanisms driving the spread of pathology in PD remain unclear. However, theories on the mechanisms of the neuronal loss include mitochondrial and lysosomal dysfunction, aberrant protein handling, oxidative stress, dopamine metabolism, and inflammation [16,17]. This is further supported by specific genes that have been implicated in genetic causes of PD, which are thought to be relevant in more than 5% of the total PD population [13]. Several genes have been identified as causative in monogenetic familial forms of PD, while others have been associated with increased risk of PD. The most common causative genes include mutations in *SNCA* (which encodes α-synuclein), *LRRK2* (which affects mitochondrial function), and *Parkin* and *PINK1* (which normally work together in the setting of mitochondrial damage) [18]. The strongest risk factor gene identified to date is *GBA* (glucocerebrosidase gene) mutations, which may involve misfolded proteins or lysosomal dysfunction and have been identified in 3–4% of PD patients compared to 1% in controls [16,19].

### 2.3. Risk Factors

In addition to genetic factors, environmental factors that confer increased risk for PD have also been identified. It is well-documented that exposure to pesticides, including rotenone, increases risk for PD. This was first proposed by the discovery that 1-methyl-4-phenyl-1,2,3,6-tetrahydopyridine (MPTP) is converted in the body into a parkinsonism-inducing molecule with a similar structure to the pesticide paraquat, which induces oxidative stress [20]. Positive associations have also been found between PD and exposure to pesticides that affect mitochondrial complex I (including rotenone) [21]. Other factors associated with increased PD risk include consumption of dairy products [16].

A pathophysiological risk factor for Parkinson’s disease is traumatic brain injury [14]. Increased attention has been given to sports-related traumatic brain injury (TBI), chronic traumatic encephalopathy (CTE) and their risk factors for development of neurological symptoms. So called pugilistic Parkinson’s and other concussion-related risk factors have been publicized due in part to Muhammad Ali’s well documented head trauma sustained over many years in boxing. Protective factors have also been identified. Many studies have demonstrated an inverse relationship between PD risk and cigarette smoking [18]. Other protective factors include caffeine consumption, physical activity, and high serum urate concentrations [14,22].

### 2.4. Presentation, Motor and Non-Motor Symptoms

Onset of PD typically occurs between the ages of 65 and 70 years [13]. However, the disease does not start suddenly with the classic motor symptoms that define PD. Many patients present with prodromal symptoms, which may be motor and/or non-motor, years before the onset of the typical parkinsonian features [16]. The most common of these include idiopathic rapid eye movement (REM), sleep behavior disorder (RBD), hyposmia, and constipation, which are especially suggestive of prodromal PD when occurring together [23]. Constipation could be related to the use of anticholinergics including benztropine and trihexyphenidyl to control extrapyramidal symptoms in PD treatment. Other prodromal markers include excessive daytime somnolence, symptomatic hypotension, erectile dysfunction, urinary dysfunction, depression, and minor motor abnormalities [24].

The most recent Movement Disorder Society (MDS) Clinical Diagnostic Criteria for PD (MDS-PD) retains motor parkinsonism as the core feature, defined as bradykinesia in combination with resting tremor, rigidity or both [25]. These features are typically asymmetric initially. As the disease progresses, spontaneous movements decrease, facial expressions diminish, speech becomes monotone, and writing often becomes small [17]. Gait changes are also noted, characterized by an asymmetric reduction in arm swing with decreased stride length and walking speed [26]. Notably, postural instability is left out of the revised MDS-PD criteria as it typically occurs in later stages of PD [25].

Non-motor symptoms are also included in the most recent MDS-PD criteria, with an absence of non-motor symptoms within five years of onset now labeled as a “red flag” against the diagnosis of PD [16]. In addition to those seen in prodromal PD, other non-motor symptoms not seen in early disease also develop as the disease progresses. Pain is the most frequent non-motor PD presentation, typically involving the motor-affected side [27]. Dysautonomia is seen in virtually all PD patients, with symptoms of orthostasis affecting nearly half [17]. Psychiatric disturbances, including depression, anxiety, and visual hallucinations, are common and can lead to paranoia, and behavioral changes [16,25]. Cognitive decline involving problems in executive function are also seen, with the diagnosis of dementia being made in approximately 50% of PD patients within 10 years [28]. It should be noted that anticholinergics used to treat PD could also contribute to the dementia seen as well.

### 2.5. PD Mimics and Differential Diagnoses

There are a variety of PD presentations and their similarity to other disorders, patients are often misdiagnosed. Important PD mimics include tremor disorders, drug-induced parkinsonism, vascular parkinsonism, Alzheimer’s disease, and Parkinson’s-plus disorders including multiple system atrophy (MSA), progressive supranuclear palsy (PSP), cortical-basal ganglionic degeneration (CBGD), and dementia with Lewy bodies (DLB). Red flags for PD mimics include lack of true parkinsonian bradykinesia, presence of major dystonia, and tremors with non-classical clinical characteristics [27]. Other features that suggest an alternative diagnosis include rapid progression of gait impairment, absence of motor symptoms, early bulbar dysfunction (dysarthria, dysphagia), severe autonomic failure within five years, bilateral symmetric parkinsonism, and lack of response to high-dose levodopa [25].

## 3. Chronic Pain

Investigation into the experience of pain has captivated medical research for decades. Despite intense focus and a number of significant advances, many aspects of the etiologies, assessment, and treatments of pain remain shrouded in mystery. This is partially because the perception of pain is diverse and transcends mere sensation, also involving complex emotional, psychological, and social elements [29,30,31]. The general approach to addressing pain involves first classifying it as either acute or chronic. Relative to chronic pain, acute pain is short lasting and occurs in close temporal proximity to an identifiable cause such as an injury or surgery. This pain usually resolves as the injured tissue heals. When pain lingers longer than the expected time for healing, it is regarded as chronic pain. Chronic pain is typically defined as pain that persists longer than 3–6 months [32,33]. Chronic pain can be the result of a discrete injury, in other words a progression of acute pain, or it can be of insidious onset with difficulty associating it to a distinct event [31]. All pain, but especially chronic pain, can be extremely distressing with debilitating impacts on individuals and society.

### 3.1. Epidemiology

Chronic pain inflicts a considerable burden both on a personal and a societal level. It is estimated that chronic pain affects 11–40% of U.S adults and approximately 20% of people worldwide [34,35]. Furthermore, 15–20% of physician visits are related to chronic pain complaints amounting to a cost of roughly € 200 billion yearly in Europe and $150 billion yearly in the United States. Even as staggering as these statistics are, many believe that these estimates are too low, and that chronic pain is actually much more pervasive. Given the enormous diversity of chronic pain syndromes, the exact prevalence of chronic pain is difficult to measure. Moreover, many chronic pain patients suffer in solitude and do not seek medical attention. Chronic pain is also often a comorbidity of other illnesses which may cause the chronic pain component to be overlooked. For example, according to the World Health Organization (WHO), unipolar depression, coronary heart disease, cerebrovascular disease, and traffic accidents will be the leading contributors to global burden of disease by 2030. Chronic pain is often a component of all of these [35,36].

Certain social and demographic factors are closely associated with chronic pain. While there is little research examining chronic pain in children and adolescents, evidence suggests that increasing age is associated with an increased prevalence of chronic pain. This is potentially explained by the fact that on average, older patients have more comorbid conditions and are more likely to have experienced noxious stimuli or injury that can trigger chronic pain. Gender also appears to play a role in chronic pain with women being more likely to be afflicted by chronic pain than men. Chronic pain exhibits an inverse relationship with socioeconomic status. Individuals living in lower socioeconomic conditions have not only been shown to have a higher prevalence of chronic pain, but also higher severity. Several clinical factors have been shown to be correlated with chronic pain. The most important of these factors is preexisting pain; that is, patients who suffer with acute or chronic pain at one site are more likely to struggle with chronic pain at another site. Patients with mental health conditions such as anxiety, depression, and catastrophizing beliefs about pain not only have a higher prevalence of chronic pain, but the pain is also more likely to be recalcitrant in nature. Obesity, sleep disorders, and a history of surgeries and medical interventions have also demonstrated relationships with chronic pain. Lifestyle factors that have shown some association with higher rates of chronic pain include smoking, alcohol abuse, low levels of physical activity, living in colder climates, and low levels of vitamin D [31,36].

### 3.2. Assessment of Chronic Pain

Regardless of etiology, the initial approach to evaluating a patient with chronic pain is similar. As with any complaint, a thorough history and physical is indispensable. The aim should be to identify and quantify important signs, symptoms and behaviors which may provide insight to possible mechanisms, help ascertain the impact on functioning, and identify targets for treatment [37]. This most often occurs at the primary care level, as primary care physicians (PCPs) are the first point of contact for the overwhelming majority of patients with chronic pain [38]. Assessment is often challenging given the complex multidimensional nature of many chronic pain syndromes, as such, multiple screening tools have been developed in order to help identify patients at risk for severe chronic pain or complications of chronic pain. For example, the STaRT Back Tool is useful in assessing the likelihood of progression from acute to chronic low back pain (LBP), the Leeds Assessment of Neuropathic Symptoms and Signs (LANSS), the Neuropathic Pain Diagnostic Questionnaire (DN4) the Neuropathic Pain Questionnaire (NPQ), ID Pain and PainDETECT are useful in the assessment of neuropathic pain, and the Hospital Anxiety and Depression Scale is helpful in chronic pain patients with associated psychosocial comorbidities [38]. Application of tools such as these can aide in assessment and help inform management of chronic pain.

## 4. Chronic Pain in Parkinson’s Disease

Resting tremor, stiffness, loss of balance, trouble speaking, and eventually, neurocognitive decline are the symptoms commonly associated with Parkinson’s disease. Lesser known, but severely contributing to the degrading quality of life in Parkinson’s, are the various manifestations of chronic pain that can present [6,10,39]. Compared to the general population of similar age, Parkinson’s patients have been confirmed to suffer from a significantly higher level and prevalence of pain [5,6]. It is important to categorize and describe this pain in Parkinson’s, as it often persists unrecognized in patients suffering from the disease who otherwise could benefit from tailored treatment [5,6]. The types of chronic pain understood to be resulting from or exacerbated in the Parkinson’s patient are musculoskeletal pain, dystonic pain, nerve pain, primary pain, akathitic pain, and gastrointestinal [39,40,41]. An overview of the types of pain and their treatments can be found summarized in Table 1.

### 4.1. Musculoskeletal Pain

The most common form of pain reported by Parkinson’s patients is of musculoskeletal origin, in 45–90% of patients [5,10,39,42]. This type of pain arises as a result of the postural changes and muscular rigidity that is seen in Parkinson’s and includes conditions such as frozen shoulder, low back pain (LBP), arthritis, and osteoporosis. Frozen shoulder can be described as a “premotor” symptom for PD, occasionally presenting in an individual within 1 or 2 years before disease onset [43]. It is a gradual decrease in range of motion accompanied with pain, insomnia, and possible atrophy of the infraspinatus [44]. Of all musculoskeletal pain seen in PD patients, back pain makes up the majority [6]. One 2018 study investigated PD patients with LBP and found lumbar arthrosis in 79.6%, scoliosis in 38.8%, and spondylolisthesis in 24.1% [45]. When scoliosis was present, it was strongly correlated with progression of PD [45]. The study noted that only a small portion of these PD patients were receiving orthopedic care respective of their condition. Secondary to back pain (71.4%) in PD is joint pain in ~52% of patients with pain, which is thought to be associated with skeletal deformation and muscle wasting [6]. Because this type of pain is common in the general population, it is difficult to say whether PD causes it, or only accentuates a preexisting condition. Loss of bone density is another characteristic of PD, possibly attributable to immobility, decreased strength and weight, or levodopa use, that poses an additional risk for fracture-related pain in a fall-risk patient [46,47]. Understanding how PD affects the musculoskeletal system is paramount in improving the quality of life for the Parkinson’s patient [6,42].

### 4.2. Dystonic Pain

Dystonia is an uncontrolled muscle contraction accompanied by a deformed posture, usually in the hands and feet of a Parkinson’s patient [48]. It causes pain and inconvenience when there is prolonged muscle contraction recurring, such as plantar flexion of the toe, which impairs simple tasks like walking or wearing shoes [49]. Studies show that 15–40% of Parkinson’s patients with pain report dystonic character [5,10]. Unlike musculoskeletal symptoms mentioned above, dystonia is rarely reported in the untreated Parkinson’s patient. Current belief is that chronic treatment with levodopa dysregulates striatal cholinergic signaling and synaptic plasticity, possibly due to epigenetic alterations taking place over time [48,50,51,52,53].

### 4.3. Peripheral and Central Neuropathic Pain

Nerve pain in PD can be subdivided into two separate etiologies. The first is radicular origin, and is presumably caused or exacerbated by the postural changes and bone deformities seen in PD [39,45]. The second can be referred to as peripheral neuropathic, and includes symptoms such as tingling, numbness, and general pain. Pathology behind the occurrence of this pain in PD is not confirmed, but it is suggested to be associated with decreased levels of vitamin B12, methylmalonic acid, and/or homocysteine due to levodopa-induced malabsorption [54,55]. Another hypothesis points toward PD as being the cause itself, with phosphorylated α-synuclein depositing in nerve fibers to produce symptoms [56]. Regardless of cause, about a quarter of Parkinson’s patients with pain report nerve pain as discussed here (20% and 31.5%), presenting an additional challenge in improving quality of life [5,10,57].

Primary, or central pain, is thought to be driven directly by PD pathology and is described to be unexplainable stabbing, burning, or scalding sensations in 4–10% of patients with pain [5,10,39,58,59]. It is known to be very painful, poorly understood, and difficult to treat [60,61]. The leading hypotheses as to the cause is some dysfunction in the pain pathways or processing of pain inputs within the central nervous system [62,63]. Concerning general chronic pain, some studies suggest that it is not the sensory perception that is modulated in PD, but the motivation to endure or avoid it that is altered [64]. This concept stems from studies that have shown dopamine, which is the neurotransmitter at loss in PD, to be involved in the emotional-motivation aspect of pain rather than the sensory-discriminative [65,66]. Additionally, it is observed that accumulation of Lewy bodies, an abnormal protein aggregate indicative of PD, occurs in the area of the brain associated with the emotional-motivational aspect of pain [67,68,69]. If this is indeed the case, then it could explain why chronic pain, especially in PD, is difficult to treat using the conventional approach to sensory-discriminative pain processing [64]. Perhaps the answer to chronic pain in PD lies in investigating the emotional-motivational aspect of pain perception [70,71].

### 4.4. Other Pain Conditions Associated with Parkinson’s Disease

Akathisia refers to an unpleasant agitating sensation that is reported in about 20% of Parkinson’s patients usually in the form of “restless legs syndrome” [72,73,74]. Although not all patients report akathisia as being a strictly painful sensation, it is known to cause insomnia and discomfort that can be treated if the health provider is able to recognize it [5]. In addition, a potential source for chronic pain are the various gastrointestinal abnormalities seen in PD. These include dysphagia, constipation, impaired gastric emptying, and problematic absorption, which could lessen the effects of oral antiparkinsonian treatments and diminishes quality of life in about half of patients with PD [75,76,77]. Pathology behind enteric symptoms appears to correlate with α-synuclein deposition and subsequent degradation of gastric innervation [78,79,80]. In the long term, this has been noted to increase susceptibility to potentially painful infections [81,82].

## 5. Treatment of Chronic Pain in Parkinson’s Disease

Effective management of chronic pain conditions is a difficult task, with up to two thirds of patients reporting dissatisfaction with their treatment results [36]. Unfortunately, PD is not exempt from this reality and achieving satisfactory outcomes when managing chronic pain in PD patients is exceptionally challenging. The extensive heterogeneity of pain that is experienced in PD patients presents obstacles in identifying targets for treatment. Furthermore, a lack of controlled studies has left a dearth of evidenced based treatment recommendations, and current regimens are largely based on case reports and empirical evidence [83,84,85]. Nevertheless, an array of pharmacological and nonpharmacological treatment options is available to attempt to relieve the chronic pain symptoms of PD patients. The treatment options are summarized in Table 2.

### 5.1. Dopaminergic Agents

Many of the clinical manifestations of PD are the result of neurodegeneration involving dopaminergic pathways in the CNS. As such, dopaminergic agents have long been a mainstay of PD treatment. The influence of dopamine specifically in PD related pain is less defined however, and thus, the role of dopamine agonism in the treatment of these symptoms is unclear [63,85,86]. There is some evidence that the basal ganglia and dopaminergic activity are related to the occurrence and perception of pain. Research in anaesthetized monkeys demonstrated that painful stimuli evokes depression in the activity of dopaminergic nigrostriatal neurons. Similarly, in rats, dopamine has been shown to exert an inhibitory effect on neuronal responses of the substantia nigra to noxious stimuli [87,88]. It would follow then that the disruption of dopamine pathways in PD may contribute to pain in the disease.

One of the most common used drugs in PD is Carbidopa-Levodopa, a combination medication that includes a dopamine precursor that is converted to dopamine in the central nervous system by the enzyme aromatic l-amino acid decarboxylase, also known as DOPA decarboxylase. Carbidopa is in a class of medications described as decarboxylase inhibitors. It works by preventing levodopa from break down before it reaches the brain, allowing for a lower dose of levodopa and causing less nausea and vomiting. Case reports have pointed to potential analgesic effects of this drug as it has been shown to lessen herpetic neuropathic pain, bone pain from breast cancer and diabetic polyneuropathy [87]. In a study conducted by Honig et al., administration of jejunal levodopa was found to significantly improve the “miscellaneous” sub score of the Nonmotor Symptoms Scale in PD patients. Pain is a component of this subscore in the Nonmotor Symptoms Scale [89]. Other dopaminergic agents have also shown potential in treatment of pain syndromes. Pramipexole, a D2 dopamine agonist, was used to effectively treat a woman with burning mouth syndrome [90]. Likewise, it decreased visual analog scale (VAS) scores in patients with fibromyalgia. This change was not significant, however, pain was a secondary outcome in this trial and therefore patients were not selected for inclusion based on their degree of pain [91]. Apomorphine, a dopamine agonist, showed efficacy in a case series in which three patients reported relief of pelvic pain and two reported relief of painful dystonic symptoms with use of the drug [92]. The drug’s analgesic properties were demonstrated again when it was found to be the only effective treatment for intractable pain in a 68 year old PD patient who had failed management with various other interventions including nerve blocks, regular analgesics, and other antiparkinsonian drugs [93]. Rascol et al. conducted a double-blind placebo-controlled study to investigate the effect of rotigotine, a dopamine receptor agonist, on PD-associated pain. Patients were treated either with a transdermal rotigotine patch or a placebo patch. At the end of 12 weeks, the study demonstrated an improvement in average pain severity over the preceding seven days in those patches receiving rotigotine, however these results did not reach significance. Significant improvements were observed in secondary outcomes such as responses to the Parkinson’s Disease Questionnaire (PDQ-8) [83]. Monoamine oxidase inhibitors (MAOI-B), such as selegiline, may also be beneficial in treating PD related pain. A post-hoc analysis revealed that safinamide reduced the number of concomitant pain treatments that PD patients relied on, and also the scores of the “bodily discomfort” domain of the Parkinson’s Disease Questionnaire-39 [84,94,95].

Perhaps the most convincing evidence for dopaminergic therapy in the treatment of chronic pain in PD is the observation that many patients report experiencing more pain during their “off states,” or times when their medication is not working optimally to control other PD related symptoms, than they do in their “on states” [63,84,85,86]. While further research is needed to help to refine the use of dopaminergic agents to treat PD related pain, this suggests that optimizing dopaminergic therapy is a valuable step.

### 5.2. Other Pharmacologic Agents

As with the dopaminergic agents, there is a paucity of literature examining other pharmacologic agents in the treatment of chronic pain in PD. Nonsteroidal anti-inflammatory drugs (NSAIDs) such as ibuprofen and diclofenac were reported by PD patients to be the medication most frequently used to treat their pain [94]. The use of these drugs has not been linked to constipation. Opioids and opioid-like medications such as oxycodone, morphine, tramadol, and codeine are also commonly employed in treatment of chronic pain in PD. These medications must be used with caution related to the risk of unpleasant neuropsychiatric and GI side effects [84,86]. A multicenter, double-blind, randomized, placebo-controlled trial showed that Targinact, a combination of the opioid agonist oxycodone and the opioid antagonist naloxone, was significantly better than placebo in delivering pain relief. The opioid antagonist in this formulation helps to minimize the unwanted adverse effects of the opioid medication [96]. Tapentadol, a central-acting oral analgesic with combined opioid and noradrenergic properties demonstrated potential as a treatment in a retrospective study of 21 PD patients. After six months of treatment, patient’s reported lower severity of pain and improved anxiety, depression, and quality of life [97]. Other drugs such as cyclooxygenase-2 (COX-2) inhibitors are also widely used [94].

Care must be taken as to not induce anticholinergic side effects when using medications including the TCAs due to additive constipation complications. [85,86]. The selective serotonin and noradrenaline reuptake inhibitors (SNRIs) also possess pain relieving and antidepressant properties, but carry less risk of anticholinergic side effects. One of these medications, duloxetine, was studied in a six-week open-label trial. A total 65% of patients reported relief with use of the medication [98]. Patients and physicians must be vigilant as there is a risk for precipitating serotonin symptom if used in combination with other treatments such as monoamine oxidase type B (MAO-B).

Localized treatments may be a useful adjunct or alternative to the aforementioned systemic treatments. Botulinum toxin (BTX) injections have been shown to be safe and effective in the treatment of various PD associated symptoms including dystonic posturing, Pisa syndrome and chronic pain [99,100,101,102]. In addition to BTX’s effect on reducing neuromuscular hyperactivity, some findings suggest that it may exert direct analgesic effects [103]. A double-blind placebo-controlled crossover study to evaluate the efficacy of BTX type A for treating pain in advanced PD began in 2014. The status of this trial is currently unclear [104].

### 5.3. Non-Pharmacologic Therapies

Deep brain stimulation (DBS) of the subthalamic nucleus (STN) has been shown in multiple studies to be effective against various types of PD associated pain symptoms. Kim et al. followed a group of PD patients scheduled for DBS. Out of 23 patients who reported pain preoperatively, they observed that DBS improved dystonic pain in 100% of patients, central pain in 92%, radicular pain in 63% and musculoskeletal pain in 61% [105]. DBS of the STN has also been demonstrated to increase pain thresholds and pain tolerance, and reduce pain-induced cerebral activity in the somatosensory cortex [106,107]. These benefits are long-lasting as pain improvement was found to endure for up to eight years [108,109,110,111]. Pain relief from DBS is likely the result of a combination of mechanisms including decreasing pathologically increased muscle tone, altering pain threshold and tolerance, and improving motor function [86]. Surgical ablation of the globus pallidus, or pallidotomy, is an alternative to DBS for PD related pain. While the literature demonstrating the efficacy of pallidotomy is not as robust as that supporting DBS, a fair amount of studies have detailed its successful use in PD related pain [87,112,113,114]. Other therapies such as repetitive transcranial magnetic stimulation (rTMS), cranial electrotherapy stimulation, and spinal cord stimulation (SCS) are being evaluated for their utility in treating PD pain symptoms [86,87]. rTMS involves repeated magnetic pulses to a brain area within a short amount of time through a stimulation coil that is placed over the scalp. Cranial electrotherapy stimulation involves giving a small amount of electric current through the head via an ear-clip electrode. Both rTMS and cranial electrotherapy stimulation are noninvasive which could make them attractive alternatives to other treatments. SCS involves the placement of percutaneous electrodes to be placed at the level of the thoracic or cervical spine which involves giving electromagnetic stimulation to the dorsal columns. More research is needed before these treatments to become common place.

### 5.4. Complementary and Alternative Medicine

In addition to pharmacologic and interventional treatments, several complementary and alternative therapies have also been investigated for alleviating PD pain. The designation of ‘alternative’ medicine is a bit of a misnomer as these treatments are typically used alongside traditional pharmacologic and surgical interventions. A number of studies have examined the benefits of complementary therapies, but there is a lack of large-scale, high-level research and results are mixed regarding their effectiveness. Fortunately, most complementary therapies are safe and well-tolerated, and current literature, though limited, does suggest a role for including these therapies as a component of a multimodal approach to pain treatment.

Massage therapy (MT) is one of the most common forms of complementary and alternative medicine [115]. Studies have shown that pressure applied during MT may stimulate vagal activity, promoting reduced anxiety and pain, as well as increasing levels of serotonin [7]. In a systematic review of 12 studies on MT for PD, Angelopoulou et al. found that MT can lessen pain, induce relaxation, and improve overall quality of life in PD patients. Specific techniques that have proven effective are classical deep therapeutic massage, traditional Japanese massage, and Thai massage [115]. Acupuncture is another technique that has been studied extensively for the treatment of pain conditions. Studies in PD patients have found that patients who received acupuncture had significant improvement in pain scores compared to controls [116].

Exercise and physical therapy, though often employed for improvement of motor symptoms in PD patients, have also been evaluated for positive effects on pain. These techniques have shown some benefits especially in improving musculoskeletal pain. In a survey study of PD patients, rehabilitative therapy and physical therapy were rated as the most effective for pain reduction, though with only temporary relief [6]. An uncontrolled study of 20 PD patients, found that a 12-week exercise program resulted in a slight, though nonsignificant improvement of pain symptoms [117]. Reuter et al. conducted a randomized controlled trial of 90 PD patients, comparing walking, Nordic walking, and flexibility and relaxation programs. The walking groups had further reduction of pain intensity as compared to a flexibility and relaxation regimen, and also had decreased numbers of patients with neck, hip and sacroiliac joint pain [118]. Yoga has been studied for patients with a wide array of neurological disorders. A recent systematic review of 94 studies concluded that yoga can be an effective adjunct to medical treatment [119]. In a study of PD patients, Myers et al. found that yoga reduced the incidence of low back pain and improved balance [120].

Finally, there has been some research into the use of cannabis for pain treatment in the setting of PD. In two small, open-label, observational trials in PD patients, cannabis was found to improve pain scores as well as significantly decrease heat and cold pain tolerance [121,122]. The authors postulated that cannabis acts on PD pain via modulation of the peripheral and central pathways [122]. While no adverse effects were found in these trials, unlike with other complimentary therapies, cannabis use carries the risk of unpleasant side effects. Patients must be aware of the potential for paranoia, delusions, breathing problems, increased heart rate, and nausea. These side effects are typically dose related with incidence increasing with higher doses.

## 6. Conclusions

With 3–5% of the elderly population currently affected, Parkinson’s disease poses an increasing threat to human health and quality of life as incidence of the disease climbs either due to chemical exposure, diet, and/or lengthened lifetime [1,11,15,20,21,123,124]. Many symptoms of the disease have been well characterized in literature, but one of special significance to those affected, chronic pain, has not, as evidenced by under-diagnosis among individuals with PD [5,6,7]. Chronic pain is of detriment to the individual, but also to society as pain inhibits productive activity and resources are sunk into management. The types of pain that may be associated with PD are substantially varied with musculoskeletal pain occurring most frequently and central pain being the least reported but most severe [5,40,61]. There is some discussion that Parkinson’s pathology has a modulating effect on pain sensation, which could amplify pain and help explain a portion of the higher incidence of chronic pain felt by Parkinson’s patients, but a definitive explanation has yet to be found [63,64,65,66,67,68,69]. Pain perception is a complicated facet of human physiology and the difficulty in managing pain reflects the limited understanding [36]. If a Parkinson’s patient is determined to suffer from chronic pain, then a treatment plan should be devised that may include dopaminergic agents, acetaminophen, NSAIDS, opioids, TCAs, SNRIs, botulinum toxin, cannabis, physical therapies, or DBS. PD has proven a very personal illness in regards to symptom presentation and treatment success varying from patient to patient. Clinical experience earned from one patient may not apply to the next. A thorough assessment of patient history and physical exam should be made in patients with Parkinson’s disease so that any incidence of chronic pain may be dealt with in the same serious and realistic manner that the visible motor symptoms are treated.

## Figures and Tables

**Table 1:** Treatment and Pain Type.

Pain Type	Treatment
Musculoskeletal	Acetaminophen NSAIDs Targinact Exercise and physical therapy Massage therapy Yoga
Peripheral and Central Neuropathic Pain	Carbidopa-Levodopa Pramipexole Apomorphine MAOI-B, like safinamide Targinact Tapentadol SNRIs DBS rTMS SCS Pallidotomy
Dystonic	Apomorphine Botulinum toxin DBS
Akatheisia	Rotigotine

**Table 2:** Treatment Options.

Pharmacologic	Interventional	Complementary & Alternative
Dopamine Agonists Anti-depressants (e.g., SSRIs, SNRIs, MAOIs-B, TCAs) Anti-convulsant (e.g., gabapentin, pregabalin) NSAIDs Opioids Botox	Deep Brain Stimulation (DBS) Spinal Cord Stimulators Repetitive trans-cranial magnetic stimulation Electrotherapy Pallidotomy	Massage Therapy Acupuncture Physical Exercise Yoga Cannabis
